# Professional Schools

**Published:** 1912-09-15

**Authors:** 


### PROFESSIONAL SCHOOLS.

A brief statistical summary of professional schools in the United States, showing number of schools, professors, students, and property, is given in the first table below. Following it is a table showing comparatively the same statistics for six periods. Reports were received from nearly all the schools, giving the number of students and graduates, and for the few schools which did not report these items the figures for the previous year were used. The figures for attendance and graduation, therefore, may be assumed to be practically correct. The totals for value of grounds and buildings, endowment, and income do not comprise all the professional schools in the United States; they are aggregates only for the schools which reported these items, and which are given in the respective detail tables. Many of the most important schools, those connected with large universities, stated that separate reports of the value of the grounds and buildings, endowment, income, and libraries could not be made, as these are used in common by different departments.

### TABLE I.

### GENERAL SUMMARY OF STATISTICS OF PROFESSIONAL SCHOOLS 1910-11.

| Class | Schools | Instructors | Students | Increase (+) or decrease (—) | Graduated in 1911 | Students having a degree. |
|---|---|---|---|---|---|---|
| Theology | 193 | 1,495 | 10,834 | — 178 | 1,877 | 3,266 |
| Law | 116 | 1,570 | 19,615 | + 48 | 3,901 | 4,180 |
| Medicine | 122 | 7,598 | 19,146 | —2,248 | 4,028 | 2,044 |
| Dentistry | 55 | 1,574 | 6,961 | + 522 | 1,764 | 122 |
| Pharmacy | 77 | 847 | 6,131 | — 95 | 1,743 | 84 |
| Veterinary med'e | 21 | 408 | 2,571 | — 146 | 706 | 18 |

| Class | Value of grounds and buildings* | Endowment funds* | Benefactions* | Total income* | Volumes in libraries* |
|---|---|---|---|---|---|
| Theology | $21,419,790 | $35,313,101 | $1,552,964 | $3,399,286 | 1,304,059 |
| Law | 3,881,350 | 1,959,969 | 76,776 | 1,178,069 | 840,208 |
| Medicine | 19,723,032 | 7,985,325 | 930,251 | 2,183,128 | 358,593 |
| Dentistry | 1,947,154 | ---------- | 10,671 | 699,204 | 31,363 |
| Pharmacy | 2,070,223 | ---------- | 14,796 | 411,311 | 75,470 |
| Veterinary med'e | 919,636 | ---------- | ---------- | 383,236 | 13,692 |

*In so far as reported.

### TABLE II.

### COMPARATIVE STATISTICS OF PROFESSIONAL SCHOOLS.

| | 1911 | 1910 | 1900 | 1890 | 1880 | 1870 |
|---|---|---|---|---|---|---|
| Theology: | | | | | | |
| Schools | 193 | 184 | 154 | 145 | 142 | 80 |
| Students | 10,834 | 11,012 | 8,009 | 7,013 | 5,242 | 3,254 |
| Graduates | 1,877 | 1,759 | 1,773 | 1,372 | 719 | ---- |
| Law: | | | | | | |
| Schools | 116 | 114 | 96 | 54 | 48 | 28 |
| Students | 19,615 | 19,567 | 12,516 | 4,518 | 3,134 | 1,653 |
| Graduates | 3,901 | 4,233 | 3,241 | 1,424 | 1,089 | ---- |
| Medicine (all classes): | | | | | | |
| Schools | 122 | 135 | 151 | 129 | 90 | ---- |
| Students | 19,146 | 21,394 | 25,213 | 15,484 | 11,929 | 6,194 |
| Graduates | 4,028 | 4,448 | 5,219 | 4,556 | 3,241 | ---- |
| Dentistry | 55 | 53 | 54 | 27 | 16 | ---- |
| Students | 6,961 | 6,439 | 7,928 | 2,696 | 730 | 257 |
| Graduates | 1,743 | 1,588 | 2,029 | 943 | 266 | ---- |
| P armacy: | | | | | | |
| Schools | 77 | 79 | 53 | 30 | 14 | ---- |
| Students | 6,131 | 6,226 | 4,042 | 2,871 | 1,347 | 512 |
| Graduates | 1,743 | 1,715 | 1,130 | 759 | 186 | ---- |
| Veterinary medicine: | | | | | | |
| Schools | 21 | 20 | 13 | 7 | ---- | ---- |
| Students | 2,571 | 2,717 | 362 | 463 | ---- | ---- |
| Graduates | 706 | 769 | 100 | ---- | ---- | ---- |

*Medical Schools.*—The reports of schools of medicine for 1910-11 indicate the continuance of progress in the direction of higher standards of general education required for admission to the study of medicine, and of the better methods of instruction, through full-time salaried professors; better laboratory and clinical facilities; and better relations with hospitals. While a few medical schools make a bachelor's degree prerequisite for admission, twenty-eight require for admission a minimum of two years of work in a standard college of liberal arts, and ten require one year of such college work. Four more announce the requirement of one year of college work, effective in 1912. The difficulties experienced by some of the medical colleges which desire to establish and enforce high standards for admission and graduation are increased by the varying requirements of State statutes and State boards of medical examiners for candidates for license to practice medicine within such States. There is a great and almost vital difference between requiring of candidates graduation from a "chartered" medical school, and requiring graduation from a "reputable" or "approved" medical school.

Over against these difficulties stands the significant co-operation of several agencies in producing a strong public sentiment favorable to more rigid professional requirements for the practice of medicine. The nation-wide agitation for more efficient State boards of health and for cordial relations between such boards and corresponding Federal agencies; and the great campaigns against tuberculosis, typhoid fever, the hookworm, and similar evils, have operated indirectly to support the efforts for improving the standards of the medical colleges. Good evidence of the efficacy of this agitation is seen in the disappearance of weaker medical schools; in the assumption by the States of fuller control than heretofore over medical education and practice within their borders; and in the significant increase in State appropriations and private benefactions to medical schools. A natural reaction in some quarters against this elevation of standards of the medical profession appears in the form of cheap and curious institutions organized to furnish short cuts to the practice of something which looks like scientific medicine. While some of these are merely narrow and queer, others, like a recently organized "university" which announces itself to be "a school of drugless medicine and bloodless surgery," could scarcely be too severely denounced. The inclusion in this report of the names and statistics of institutions whose standards are at least highly uncertain, if the institutions themselves are not actually fraudulent, is obviously impossible.

One proposal for improvement of standards is the addition of a fifth, or clinical year to the four now generally required for the degree in medicine. While some institutions have preferred to strengthen the foundations of the course, others have been earnestly discussing the addition of a fifth year at the end of the usual course. It is interesting to note that the students themselves seem to have but one opinion as to the desirability of a year of practical experience in a hospital. Though they desire to complete their professional preparation in as short time as possible, they estimate so highly the value of hospital experience that they compete eagerly for appointments to hospital service. The statistical information collected by this office shows that already a large proportion of the graduates of the better medical schools secure such appointments for one or two years. The requirement of an additional year, therefore, would not impose serious hardship upon many students, since the additional expense of such a year, in time and money, is now voluntarily assumed by the great majority of graduates from strong medical schools.

*Increase of Benefactions for Medical Education.*—Benefactions for the endowment and support of medical education reported for 1910-11 amount to $930,251. This indicates a movement among givers to make medical education and research objects of special favor. The alliance between hospitals and medical colleges appears to grow closer each year. Several publicly supported hospitals have placed their entire control and management under the direction of the faculties of medical schools. It is now quite true that a large percentage of the sums given for endowment and support of hospitals contributes directly to the improvement of medical education.—*Reprint from Report of the Commissioner of Education for June 30, 1911.*

